# Ecological Distribution and CQ11 Genetic Structure of *Culex pipiens* Complex (Diptera: Culicidae) in Italy

**DOI:** 10.1371/journal.pone.0146476

**Published:** 2016-01-07

**Authors:** Marco Di Luca, Luciano Toma, Daniela Boccolini, Francesco Severini, Giuseppe La Rosa, Giada Minelli, Gioia Bongiorno, Fabrizio Montarsi, Daniele Arnoldi, Gioia Capelli, Annapaola Rizzoli, Roberto Romi

**Affiliations:** 1 Department of Infectious, Parasitic and Immune-Mediated Diseases, Istituto Superiore di Sanità, viale Regina Elena, 299, 00161, Rome, Italy; 2 National Centre for Epidemiology, Surveillance and Health Promotion, Istituto Superiore di Sanità, viale Regina Elena, 299, 00161, Rome, Italy; 3 Laboratory of Parasitology - Istituto Zooprofilattico Sperimentale delle Venezie, viale dell’Università 10, 35020, Legnaro, Padua, Italy; 4 Department of Biodiversity and Molecular Ecology, Research and Innovation Centre, Fondazione Edmund Mach, Via E. Mach 1, 38010, San Michele all'Adige, Trento, Italy; University of Camerino, ITALY

## Abstract

Mosquitoes in the *Culex pipiens* complex are considered to be involved in the transmission of a range of pathogens, including West Nile virus (WNV). Although its taxonomic status is still debated, the complex includes species, both globally distributed or with a more limited distribution, morphologically similar and characterised by different physiological and behavioural traits, which affect their ability as vectors. In many European countries, *Cx*. *pipiens* and its sibling species *Culex torrentium* occur in sympatry, exhibiting similar bionomic and morphological characters, but only *Cx*. *pipiens* appears to play a vector role in WNV transmission. This species consists of two biotypes, *pipiens* and *molestus*, which can interbreed when in sympatry, and their hybrids can act as WNV-bridge vectors, due to intermediate ecological features. Considering the yearly WNV outbreaks since 2008 and given the morphological difficulties in recognising species and biotypes, our aim was to molecularly identify and characterised *Cx*. *pipiens* and *Cx*. *torrentium* in Italy, using recently developed molecular assays. *Culex torrentium* was not detected; as in other European countries, the *pipiens* and *molestus* biotypes were widely found in sympatry with hybrids in most environments. The UPGMA cluster analysis applied to CQ11 genotypic frequencies mainly revealed two groups of *Cx*. *pipiens* populations that differed in ecological features. The high propensity of the *molestus* biotype to exist in hypogean environments, where the habitat’s physical characteristics hinder and preclude the gene flow, was shown. These results confirmed the CQ11 assay as a reliable diagnostic method, consistent with the ecological and physiological aspects of the populations analysed. Since the assessment of the actual role of three biotypes in the WNV circulation remains a crucial point to be elucidated, this extensive molecular screening of *Cx*. *pipiens* populations can provide new insights into the ecology of the species and may give useful indications to plan and implement WNV surveillance activities in Italy.

## Introduction

Mosquitoes in the *Culex pipiens* complex are considered to be involved in the transmission of a range of pathogens, including West Nile virus (WNV, family *Flaviviridae*, genus *Flavivirus*), responsible for a febrile (WND) and a neuro-invasive disease (WNND) that can affect horses and humans [1–2].

The taxonomy and phylogeny of the *Cx*. *pipiens* complex remains controversial among specialists, due to the difficulty in clearly discriminating all members at the morphological level. The complex includes two widespread mosquitoes–*Culex pipiens* Linnaeus, 1758 and *Culex quinquefasciatus* Say, 1823 –which are vector species in temperate and tropical regions of the world, respectively, as well as two other species–*Culex australicus* Dobrotworsky & Drummond 1953 and *Culex globocoxitus* Dobrotworsky, 1953,–whose distributions are limited to Australia [3–5]. *Culex pipiens* has two recognised subspecies, *Cx*. *pipiens pipiens* and *Culex pipiens pallens* Coquillett, 1898, which occur in temperate Asia. Furthermore, *Cx*. *p*. *pipiens* has two epidemiologically distinct forms or biotypes, *pipiens* and *molestus*, which differ dramatically in a number of behavioural and physiological characteristics that affect their vector competence for WNV. The *pipiens* biotype, the rural form, mates in outdoor swarms (eurygamous) and requires a bloodmeal for egg development (anautogenous), it bites mostly birds (ornithophilic), oviposits in open-air habitats (epigeous) and undergoes hibernation as gravid females (heterodynamic). The *molestus* biotype, the urban form, does not require large spaces for adult swarming or mating (stenogamous) and lays at least the first batch of eggs without a bloodmeal (autogenous), although it can bite mammals and in particular humans readily (anthropophilic), it oviposits in enclosed habitats (hypogeous) and does not diapause, remaining active during the winter (homodynamic) [6–8].

A closely related sibling species, *Culex torrentium* Martini, 1925, which is morphologically very similar to members of the *Cx*. *pipiens* complex, is commonly confused with *Cx*. *pipiens*. Both species occur in sympatry throughout Europe [4,5,9] and are potential vectors of arboviruses, but only the nominal species appears to play a primary role in the maintenance, amplification, and transmission of WNV in Europe, both in rural and urban ecosystems [10–14].

As WND impacts on European countries every year, including Italy since 2008, it is now considered to be one of the major causes of public health concern in this area [14–15]. Consequently, the discrimination of vector species and the evaluation of their involvement in virus circulation is becoming an important issue for WND risk assessment and for the adoption of correct public health strategies [16].

The identification of *Cx*. *pipiens* complex members and other sibling species, such as *Cx*. *torrentium*, relies on the morphology of the male genitalia (phallosoma) [17], excluding *de facto* mosquito females, which mainly represent the target of surveillance and control efforts. Only the prealar scales permit females of *Cx*. *pipiens* and *Cx*. *torrentium* to be discriminated [17], but this key trait is easily rubbed off during the collection and the handling of mosquitoes. Furthermore, hybrids among *Cx*. *pipiens* complex species often show intermediate characters and no morphological traits exist to distinguish between the two biotypes of *Cx*. *pipiens* [6].

To circumvent these difficulties, molecular assays to differentiate *Cx*. *pipiens* and *Cx*. *torrentium* or to distinguish between the *Cx*. *pipiens* forms have been developed and implemented for mosquito populations in the Palearctic region [18–29].

Although the accurate distribution of both *Culex* species is largely unknown, *Cx*. *torrentium* certainly dominates central and northern Europe at latitudes below 48°N [30–32], although there are previous records of species from southern countries, including Italy [33–34].

It is known that the sympatry of the two *Cx*. *pipiens* forms appears to be a common condition in several southern European countries and in North Africa [22,25,28–29,35–36]. In such circumstances, *molestus* and *pipiens* biotypes can interbreed and their hybrids, which exhibit intermediate ecological features, can act as WNV-bridge vectors, as was shown during outbreaks in the United States [35,37–38] and confirmed through WNV experimental infections [39]. In northern Palearctic latitudes, the two forms occur in distinct habitats and show different ecological features that completely hinder the gene flow [9,30,40–41]. Nevertheless, the recent detection of molecular hybrids reported for the Netherlands, Germany and the United Kingdom appears to contradict this thesis [23,26–27].

In the light of these studies, we aimed to molecularly determine the presence of *Cx*. *pipiens* and *Cx*. *torrentium* in 55 localities in Italy and to subsequently investigate their behavioural and physiological features by acquiring data from field populations and from laboratory colonies. To identify *Cx*. *pipiens* forms and their hybrids, we tested two recently developed molecular assays based on the CQ11 [19] and COI [20] loci as diagnostic markers, whose reliability has been debated [26,42] and was herein also evaluated.

## Materials and Methods

### Ethics Statement

No specific permits were required for the field studies. All field mosquito populations were collected from public areas. No sites were protected by law and this study did not involve endangered or protected species.

The protocol for routine blood mosquito feeding has been approved by the Service for Biotechnology and Animal Welfare of the Istituto Superiore di Sanità (National Institute of Health) and has been authorised by the Italian Ministry of Health with the Decree 222/2011-B, according to the Legislative Decree 116/92, which implemented in Italy the European Directive 86/609/EEC on laboratory animal protection. The animals used in this study were housed and treated in strict accordance with the recommendations in the Legislative Decree 116/92 guidelines and animal welfare was routinely checked by veterinarians from the Service for Biotechnology and Animal welfare. In particular, 30 female hamsters (*Mesocricetus auratus*) per year were used to maintain all mosquito colonies in Insectary and each hamster was housed in a single plastic shoe-box cage (26x20x14 cm). The husbandry protocol provided Lignocel^®^ Select-Fine as commercial dust-free bedding with a replacement of the bedding materials routinely done twice weekly; a standard pellet diet (Altromin-7024, Rieper, Vandoies, Italy) and water were supplied *ad libitum*. The animals were daily monitored by animal technicians and weekly examined by a veterinarian. Before blood feeding, the selected hamster was anesthetized, using Ketamine/Xylazine combination as anesthetic. A continuous rotation of all hamsters was planned to allow a complete recovery, after every use. Euthanasia of each hamster was considered after 6–8 mosquito blood meals by an overdose of anesthetic.

### Mosquito collection

The *Culex* mosquitoes were collected in 55 discrete localities in Italy from 2004 to 2014. The collection sites were defined by habitat (urban, peri-urban, rural or natural) and by breeding site (aboveground or underground), when found (Table 1). In particular, the habitats were classified as urban fabric (artificial surfaces with a dominance of urbanised areas), rural (areas devoted to agriculture) or natural (forests, wetlands and natural parks in which human activities were limited or absent), according to CORINE land-cover nomenclature [43]. The urban fabric was further categorised as urban (high-density housing and commercial areas with >80% of the total surface covered by buildings and roads and a human density exceeding 300 inhabitants per km^2^) or peri-urban (low-density housing with a discontinuous urban structure covering between 30 to 80% of the total surface and a human density < 300/km^2^) [43, 44].

**Table 1:** Characteristics of *Culex pipiens* sites sampled in Italy. Mosquito collection sites with the respective identification number (ID) and number of *Culex pipiens* individuals analysed with reference to habitat, breeding site and collection date.

ID	Locality (Province)	Number of specimens analysed	Habitat	Breeding site	Collection date	Latitude	Longitude
**1**	Monticolo Lake (BZ)	20	Rural	Above	July 2014	46°26'31"N	11°16'28"E
**2**	Ponte Oliveti (TN)	6	Rural	Above	Aug–Sept 2011	46°2'34"N	10°57'42"E
**3**	Ceniga (TN)	11	Rural	Above	Aug–Sept 2011	45°57'2"N	10°53'59"E
**4**	Arco (TN)	14	Urban	Above	Aug–Sept 2011	45°55'4"N	10°53'12"E
**5**	Bolognano (TN)	7	Rural	Above	Aug–Sept 2011	45°54'49"N	10°54'14"E
**6**	Riva del Garda (TN)	12	Urban	Above	Aug–Sept 2011	45°53'17"N	10°50'40"E
**7**	Meolo (VE)	19	Rural	Not found	July 2009	45°37'12"N	12°27'9"E
**8**	Caorle (VE)	20	Peri-urban	Not found	Sept 2009	45°35'41"N	12°52'14"E
**9**	Gazzo Padovano (PD)	20	Rural	Above	July 2009	45°33'30"N	11°40'47"E
**10**	Mira (VE)	20	Peri-urban	Above	July 2009	45°25'24"N	12°9'10"E
**11**	Valle Averto Oasis (VE)	20	Natural	Above	Sept 2010	45°21'20"N	12°5'46"E
**12**	Legnaro (PD)	10	Peri-urban	Above	Sept 2013	45°20'34"N	11°58'5"E
**13**	Brugine (PD)	20	Peri-urban	Above	Sept 2009	45°17'51"N	11°59'46"E
**14**	S. Anna di Chioggia (VE)	11	Rural	Above	Aug 2004	45°9'1"N	12°16'13"E
**15**	Rosolina (RO)	19	Rural	Above	Aug 2004	45°8'9"N	12°19'3"E
**16**	Cavarzere (VE)	17	Peri-urban	Not found	July 2009	45°8'4"N	12°4'51"E
**17**	Trecenta (RO)	20	Peri-urban	Not found	July 2009	45°1'55"N	11°27'39"E
**18**	Papozze (RO)	18	Rural	Above	July 2009	44°59'12"N	12°1'55"E
**19**	Ficarolo (RO)	17	Rural	Above	Sept 2009	44°57'19"N	11°26'9"E
**20**	Pomposa (FE)	8	Rural	Above	Aug 2004	44°50'20"N	12°10'34"E
**21**	Ravenna (RA)	20	Rural	Above	May 2004	44°24'56"N	12°11'47"E
**22**	Sala di Cesenatico (FC)	16	Rural	Above	May 2005	44°9'20"N	12°23'8"E
**23**	Villa Verrucchio (RN)	20	Rural	Above	July 2006	44°0'18"N	12°26'7"E
**24**	Borgo a Buggiano (PT)	38	Rural	Above	Sept 2007	43°52'46"N	10°43'40"E
**25**	Galleno (PI)	10	Rural	Above	Sept 2007	43°46'32"N	10°43'14"E
**26**	Torre Matteucci alle Paludi (FM)	23	Rural	Not found	Aug–Sept 2012	43°9'46"N	13°43'5"E
**27**	Lido di Fermo (FM)	7	Urban	Not found	Aug 2014	43°12'0"N	13°47'23"E
**28**	Castiglion del Lago (PG)	20	Rural	Above	July 2013	43°7'41"N	12°2'46"E
**29**	Principina (GR)	6	Rural	Above	July 2007	42°43'29"N	11°2'28"E
**30**	Narni (TR)	20	Peri-urban	Above	Apr 2014	42°31'31"N	12°30'51"E
**31**	Montelibretti (RM)	20	Urban	Under	Mar 2012	42°8'53"N	12°38'44"E
**32**	Avezzano Cemetery (AQ)	14	Urban	Above	Aug 2011	42°1'34"N	13°25'31"E
**33**	Guidonia (RM)	14	Urban	Above	Sept 2010	41°59'59"N	12°43'34"E
**34**	Cerveteri (RM)	11	Rural	Not found	Sept 2010	41°59'37"N	12°5'36"E
**35**	Subiaco (RM)	18	Urban	Under	July 2011	41°55'35"N	13°5'42"E
**36**	Castel di Guido (RM)	20	Rural	Above	Aug 2009	41°54'11"N	12°17'1"E
**37**	Rome—Insugherata Park (RM)	16	Natural	Above	Sept 2012	41°57'27"N	12°25'39"E
**38**	Rome—RAI (RM)	9	Urban	Under	May 2012	41°55'54"N	12°27'27"E
**39**	Rome—Verano Cemetery (RM)	32	Urban	Above	July 2004	41°54'10"N	12°31'30"E
**40**	Rome—Aurelio (RM)	20	Urban	Above	Sept 2009	41°53'55"N	12°24'48"E
**41**	Rome—Valcannuta (RM)	18	Urban	Under	Apr 2014	41°53'55"N	12°25'2"E
**42**	Rome—Forlanini Hospital (RM)	10	Urban	Under	Oct 2012	41°51'58"N	12°27'1"E
**43**	Rome—Caffarella Park (RM)	10	Urban	Above	Nov 2012	41°51'49"N	12°31'9"E
**44**	Rome—Magliana (RM)	20	Urban	Above	July 2007	41°49'22"N	12°23'23"E
**45**	Frascati (RM)	19	Rural	Above	Aug–Sept 2009	41°48'22"N	12°40'49"E
**46**	Anagni (FR)	16	Rural	Above	Sept 2012	41°44'47"N	13°9'3"E
**47**	Manfredonia (FG)	12	Rural	Above	July 2013	41°37'45"N	15°54'47"E
**48**	Fogliano Lake (LT)	17	Rural	Above	May 2012	41°24'5"N	12°54'28"E
**49**	Terracina (LT)	18	Rural	Above	Sept 2009	41°17'28"N	13°14'55"E
**50**	Ischia Isle (NA)	23	Peri-urban	Not found	July 2014	40°43'46"N	13°55'31"E
**51**	Matera (MT)	15	Rural	Above	July 2013	40°39'36"N	16°36'5"E
**52**	Scalea (CS)	6	Peri-urban	Above	Sept 2011	39°48'52"N	15°47'27"E
**53**	Rende (CS)	12	Urban	Above	Sept 2011	39°19'53"N	16°10'53"E
**54**	Zafferana (CT)	47	Peri-urban	Above	June 2013	37°39'29"N	15°7'13"E
**55**	La Maddalena Isle (OT)	8	Peri-urban	Above	Aug 2007	41°13'57"N	9°25'28"E

Mosquitoes were sampled as adults, using CO_2_-baited miniature light traps from the US Centers for Disease Control and Prevention (Atlanta, GA, USA) or BG Lure^®^-Baited Biogents Sentinel Traps and as immatures, using the dipping sampling method. Larvae and pupae were reared to adulthood in an insectary (26 ± 1°C; 70 ± 10% RH, and a light:dark cycle of 16:8 h), with a larval mortality ranging between 5% and 10%. Mosquitoes were morphologically identified as *Cx*. *pipiens/Cx*. *torrentium* according to Severini *et al*. [45] and were stored at -20°C until molecular processing.

A long-established laboratory-reared colony (hereinafter cited as ISS-colony) and several wild *Cx*. *pipiens* populations (ID 9, 30, 31, 38, 41, 42, 43, 45 and 54), were reared in an insectary for several filial generations (ranging from F2 for ID 9 to F31 for ID 45), to evaluate mating and autogenic behaviour. Immatures were bred in a 3‰ sodium chloride solution and were supplemented with fish flakes as food. Emerging male and female mosquitoes were bred in cages (26 cm sides; 0.017 m^3^) with access to a 10% sucrose solution. To monitor autogenic behaviour, an oviposition tray was kept in each cage and was observed daily for 15–20 days. After this period, bloodmeal supply was provided to lay anautogenous egg rafts.

### Molecular analyses

The DNA from individual *Culex* specimens was extracted using the PureLinkTM Genomic DNA Mini Kit (Invitrogen, Carlsbad, CA, USA) according to the manufacturer’s protocol.

Mosquitoes were molecularly identified as *Cx*. *pipiens* or *Cx*. *torrentium* by a multiplex PCR based on a polymorphism in the second intron of the acetylcholinesterase gene (ACE-2 assay) [18]. A second multiplex PCR was subsequently used to detect a polymorphism in the flanking region of the CQ11 microsatellite of *Cx*. *pipiens* specimens, which generated a 190-bp amplicon in the *pipiens* form, a 260-bp amplicon in the *molestus* form, and both PCR products in hybrids of both forms [19].

Eighty-eight individuals from eight *Cx*. *pipiens* populations (ID 9, 10, 13, 36, 37, 39, 45 and the ISS-colony), previously identified by the ACE-2 and CQ11 assays and characterised by breeding sites (hypogean/epigean) and anauto-/autogenic behaviour, were further analysed using a RFLP-PCR of the COI gene [20]. This method (hereinafter cited as the COI assay) discriminates individual specimens of the *molestus* and *pipiens* biotypes and *Cx*. *torrentium*, using restriction sites for *Hae*III and *Bc*II of mtDNA COI gene. In addition, 26 out of 88 COI amplicons were sequenced and compared with the GenBank sequences from Russian mosquitoes: *Cx*. *pipiens* form *molestus* (AM403492), *Cx*. *pipiens* form *pipiens* (AM403476) and *Cx*. *torrentium* (AM403477). The sequences herein generated are available in GenBank under the following accession numbers: KP728846–KP728871.

DNA samples from nine *molestus* specimens belonging to an autogenous colony (purchased from Bioagents AG, Germany), from two *pipiens* specimens and from thirteen *Cx*. *torrentium* specimens (kindly offered by Dr. J. C. Hesson, Sweden), were used as internal controls.

### CQ11 population analysis

The existence of gene flow between *pipiens* and *molestus* biotypes was investigated by verifying the Hardy–Weinberg equilibrium (HWE) using the CQ11 locus; if gene flow occurs between the two forms, the frequencies of the CQ11 alleles should not show a significant departure from the HWE. An exact test for the HWE was restricted to 24 largest localities for which the sample size was more than 18 (ID 7, 8, 9, 10, 11, 13, 15, 17, 18, 21, 23, 24, 26, 28, 30, 35, 36, 39, 40, 44, 45, 49 50 and 54) and computed by Genepop ver. 4.0 [46]. The inbreeding coefficient (Fis) [47] was computed in Genepop ver. 4.0 and the significance of the Fis values was analysed using FSTAT ver. 2.9.3 [48]. The CQ11 genetic relationship between *molestus* and *pipiens* populations was studied using the Nei 72 genetic distance and UPGMA algorithm of clustering as implemented in Populations ver.1.2.32 software [49].

### Statistical analysis

To test whether the distribution of biotypes of each *Cx*. *pipiens* population was significantly associated with the habitat and breeding site, a multinomial logistic regression was performed using SPPS software (version 22). The biotype composed of three categories, *molestus*, hybrid and *pipiens* (the reference category), was selected as the dependent variable, and the habitat and breeding site as independent variables. A Chi-squared test/Fisher’s Exact test were used to assess the percentages of *pipiens*, hybrid and *molestus* biotypes from the colony (from ID 45) in each filial generation. To evaluate the composition of each biotype during the selected filial generations, the significance was tested using a non-parametric test for trends across the ordered groups (nptrend command in STATA [50]). All statistical tests were considered significant at the *p* ≤ 0.05 probability level.

## Results

Overall, 914 *Cx*. *pipiens* specimens were collected in 55 localities from 14 out of 20 Italian regions. All specimens were molecularly typed using ACE and CQ11 PCR at the biotype level.

### ACE, CQ11 and COI identification

*Culex torrentium* was not identified by PCR in this study.

**Fig 1:**
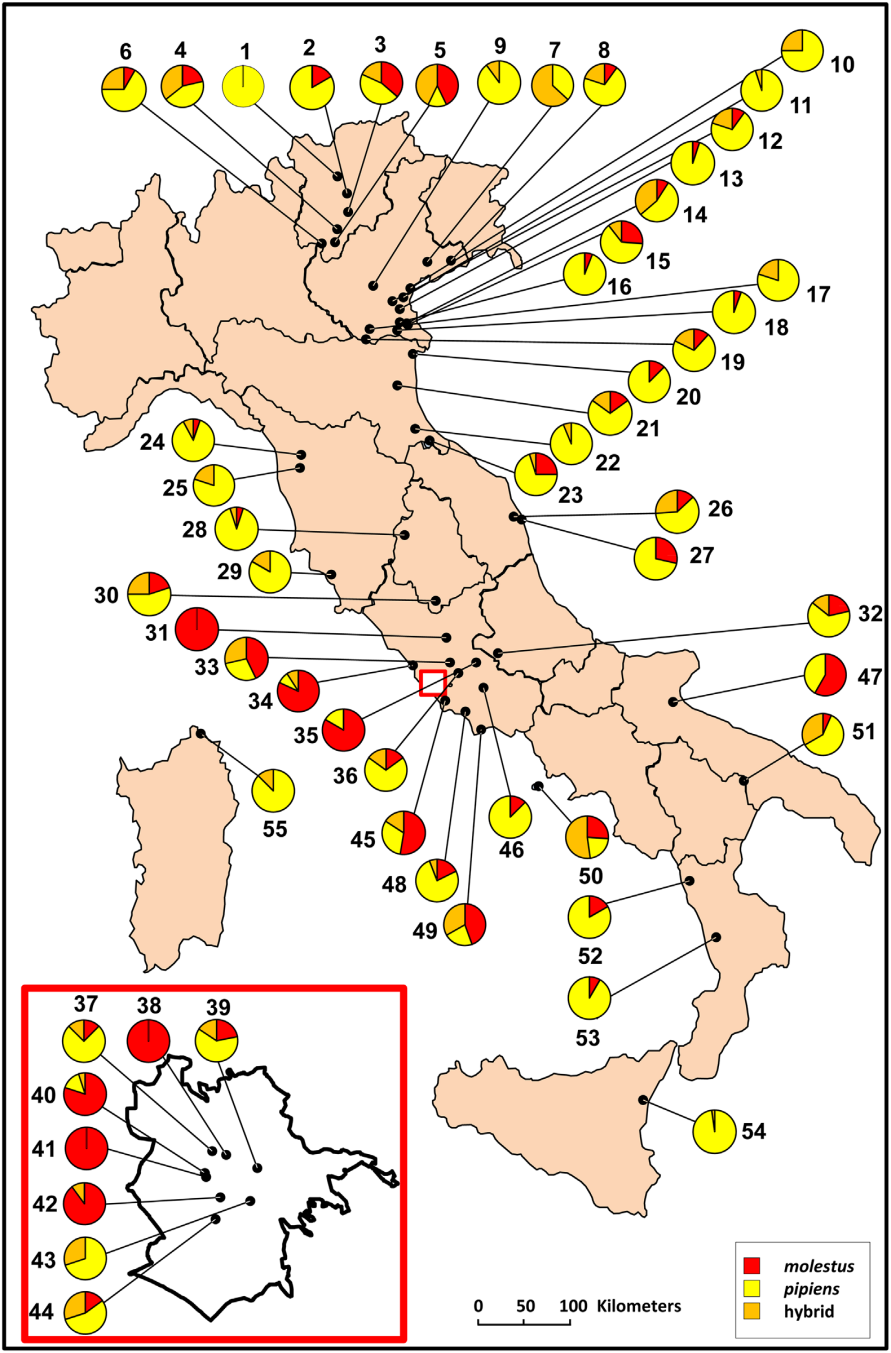
Distribution of *Culex pipiens* in Italy. Composition of the *Culex pipiens* genotypes of 55 field-collected populations in Italy using the CQ11 assay.

Different frequencies of CQ11 genotypes in *Cx*. *pipiens* populations were observed in all localities (Table 1 and Fig 1).

Out of the total number of analysed specimens, 576 (63.0%) were identified as the *pipiens* form, 206 (22.6%) as the *molestus* form, and the remaining 132 (14.4%) as hybrids. Overall, 28 (50.9%) out of the 55 populations were screened for sympatric presence of *Cx*. *pipiens* biotypes and their hybrids were observed at different frequencies, whereas pure populations were extremely rare, with only one of *pipiens* (1.8%; ID 1) and three of *molestus* (5.5%; ID 31, 38 and 41) being present. Eleven *Cx*. *pipiens* populations (20%) were characterised by the two parental biotypes and no hybrids were observed; 11 populations (20%) shared the hybrid and *pipiens* forms and hybrids were found with *molestus* specimens only in one population (1.8%; ID 42).

Statistical analysis showed a higher propensity of the biotype *molestus* to exist in underground foci (e^β^ = 7.68; *p* < 0.001), mainly within urban environments (e^β^ = 2.82, but this state was not significant; *p* < 0.2), with respect to *pipiens* biotype. The *molestus* populations (ID 31, 38 and 41) were found only in urban settings, in flooded foundations of buildings as a breeding site, with no or very limited access to the outside environment. A similar context was observed in the underground tufa-caves (7,000 m^2^ wide and about 10 m high) of ID 42 (Forlanini hospital complex). This habitat, which harbours a subterranean lake (about 40 m in diameter) that is connected to the outside through a long tunnel, was steadily filled with freshwater from an aquifer and had a constant temperature of 13°C throughout the year. The genotyping of the larvae collected at the site showed the presence of 90% of *molestus* and 10% of hybrids. In contrast, the flooded basements of ID 35 (hospital of Subiaco), which were closely connected to the outside, harboured a population containing 83% of *molestus* and 17% of *pipiens* biotypes.

However, *molestus* specimens were also found in aboveground populations living in natural and rural areas [ranging from 5% (ID 13) to 82% (ID 34)]. Hybrid forms were found to be equally distributed in both above- and underground environments (*p* < 0.001 and *p* = 0.023, respectively). Although only one pure population of *pipiens* biotype was found (ID 1), this form was observed in a further 50 populations (92.7%), thriving mainly in aboveground breeding sites.

To compare two available molecular methods that are widely used to discriminate the *Cx*. *pipiens* biotypes, 88 specimens belonging to seven aboveground populations and to one long-established autogenous *Cx*. *pipiens* colony were analysed using both CQ11 and COI assays (Table 2).

**Table 2:** Comparative molecular identifications of a *Culex pipiens* subset. Eighty-eight *Culex pipiens* specimens from seven Italian localities (ID) and from an ISS-colony were tested for CQ11 and COI assays. P = *pipiens*, M = *molestus* and M/P = CQ11hybrid.

ID	Locality	Number of mosquitoes assayed for both molecular targets	COI assay		CQ11 assay		
			P	M	P	M/P	M
9	Gazzo Padovano	5		5	5		
10	Mira	19		19	14	5	
13	Brugine	5		5	4		1
36	Castel di Guido	14	14		9	2	3
37	Rome-Insugherata Park	8	8		4		4
39	Rome-Verano	9	9		2	2	5
45	Frascati	17	17		5	3	9
	ISS-colony	11	11		1	2	8

As expected, both methods allowed individuals to be separated into two forms, recognised as *pipiens* and *molestus*, but only the CQ11 assay identified a third double-banded pattern defined as hybrids.

The analysis of 23 specimens from ID 9, 10 and 13, recognised as *pipiens* by the CQ11 assay, were identified as *molestus* by the COI assay; 21 samples from ID 36, 37, 39 and 45 identified as *molestus* by the CQ11 assay, showed a *pipiens* pattern by the COI assay. All five specimens from ID 10 that were identified as hybrids by CQ11 were identified as *molestus* by the COI assay, whereas the remaining seven hybrid individuals from ID 36, 39 and 45 were identified as *pipiens*. All 11 specimens of the ISS-colony identified as *pipiens* (N = 1), *molestus* (N = 8) and hybrids (N = 2) by CQ11, showed a *pipiens* banding pattern in the COI assay.

The COI locus was amplified for 26 of the 88 mosquitoes analysed using both methods and the 603 bp amplicon was sequenced (Table 3).

**Table 3:** Fraction of *Culex pipiens* specimens sequenced for COI. After the CQ11 and COI analyses, 26 *Culex pipiens* specimens from ID 9, 10, 13 and from the ISS colony were further sequenced for the COI gene (GenBank accession numbers: KP728846-KP728871), confirming the apparent incongruity between the two assays (see text). P = *pipiens*, M = *molestus* and M/P = CQ11 hybrid.

ID	Locality	Proportion of COI sequences out of the mosquitoes double analysed	COI sequencing		CQ11 assay		
			P	M	P	M/P	M
9	Gazzo Padovano	5/5		5	5		
10	Mira	5/19		5	4	1	
13	Brugine	5/5		5	4		1
	ISS colony	11/11	11		1	2	8

The results showed that five specimens from ID 9 (KP728846-KP728850), five from ID 10 (KP72885-KP728855) and five from ID 13 (KP728856-KP728860) shared 100% identity with the *molestus* biotype from Russia (AM403492). Conversely, for 11 specimens of the ISS-colony (KP72886-KP728871), the COI-sequences showed 99.8% identity with the *pipiens* biotype from Russia (AM403476), differing only at position 292 (G to A).

### CQ11 population analysis

A significant HWE departure (p < 0.05) was observed for 11 localities (46%) out of 24 that had a sample size greater than 18 and where both *pipiens* and *molestus* biotypes were found or supposed by the presence of heterozygotes. Hence the presence in these localities of two separate gene pools can be supposed. In three of these populations (ID 13, 18 and ID 35) the presence of both homozygotes but no heterozygotes further support this hypothesis. Taking in account all 55 localities, the absence of heterozygotes in presence of both homozygotes was observed in 11 of them.

Out of the 24 populations for which the HWE significance was computed, 19 (79%) showed a significant heterozygote deficit (positive Fis values, *p* < 0.05). Significant positive Fis values were not observed for the others 31 localities.

**Fig 2:**
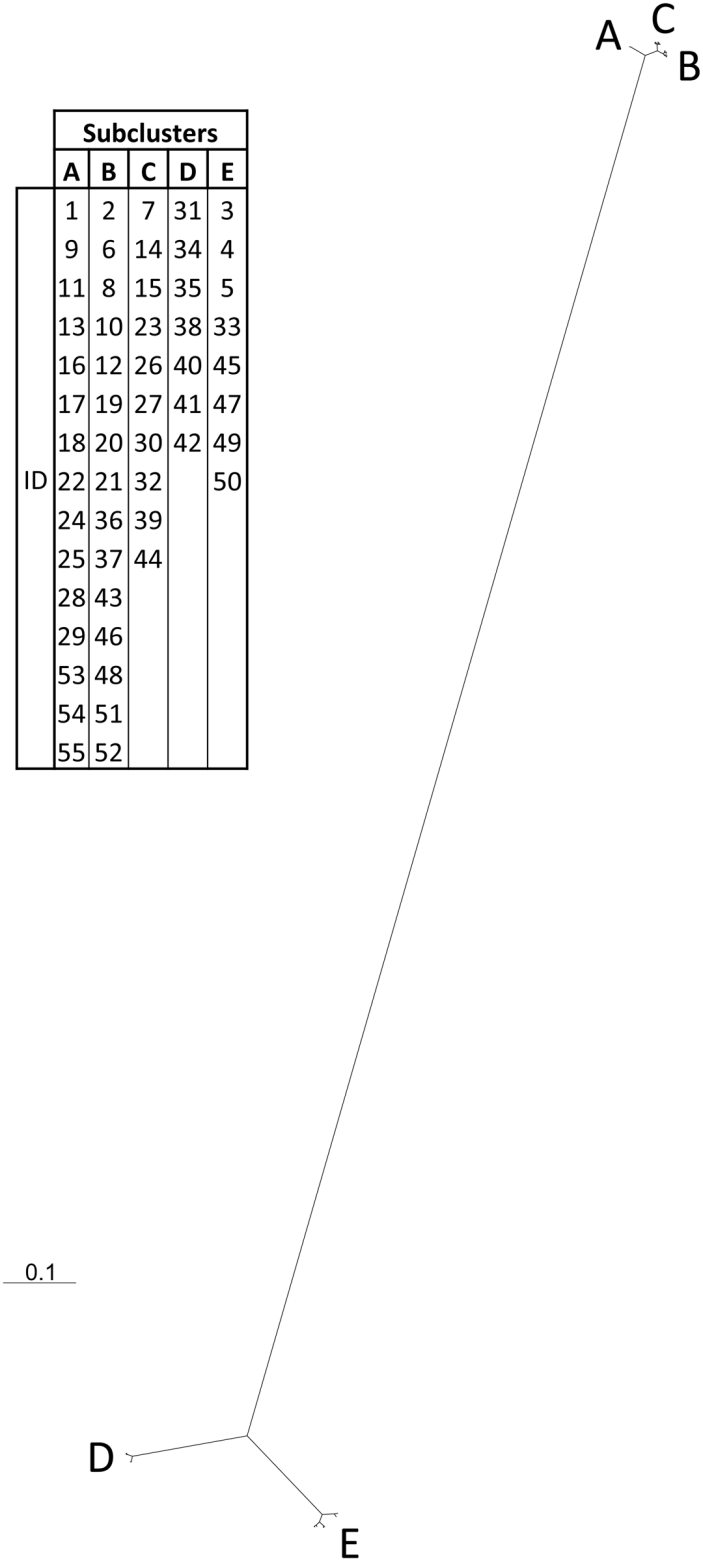
Ecological and genetic relationships among Italian *Culex pipiens* populations. The dendrogram evaluated the *Culex pipiens* population genotype frequencies by UPGMA cluster analysis, based on the Nei 72 algorithm.

The relationship among clusters as depicted by CQ11 locus analysis, is visualised in Fig 2, where UPGMA cluster analysis (based on the Nei 72 algorithm) clearly identified two distinct main assemblages, which were ungrouped by geographic distribution, but rather grouped by ecological characters.

In the first main cluster composed of 40 *Cx*. *pipiens* populations, three sub-clusters A, B and C are recognisable. Cluster A is characterised by 15 populations with higher frequencies of the CQ11^190/190^ genotype (from 80 to 100%), and represents aboveground mosquito foci and adult collection sites that were mainly located in natural, rural and peri-urban environments. The other two sub-clusters, B and C, which were more closely related to each other than to the sub-cluster A, showed *pipiens* genotype frequencies ranging from 60 to 87.5% and from 36.8 to 71.4%, respectively, which represented aboveground breeding sites and adults found in natural, rural, peri-urban, but also urban environments. The second main cluster was composed of 15 *Cx*. *pipiens* populations grouped in two distinct sub-clusters, D and E. Sub-cluster D, shared by seven *Cx*. *pipiens* populations with frequencies of the CQ11^260/260^ genotype ranging from 80 to 100%, was mainly characterised by an urban habitat and underground breeding sites. Sub-cluster E included eight *Cx*. *pipiens* populations with intermediate frequencies of the three genotypes (21.4–58.3% for CQ11^260/260^; 14.3–45.5% for CQ11^190/190^ and 0–52.2% of CQ11^190/260^), with adults collected in rural, peri-urban and urban habitats and immatures developing in aboveground breeding sites.

### Analysis of offspring

Nine *Cx*. *pipiens* populations (ID 9, 30, 31, 38, 41, 42, 43, 45 and 54) collected from both above- and underground habitats, were established and reared in insectary conditions for several filial generations, to acquire phenotypic and physiological data (i.e., mating and autogenic behaviour) to be related with genotyping.

Insemination behaviour was observed in all these natural populations, which showed the ability to mate in cage conditions. Both the ID 9 and 54 populations (CQ11 genotyped in the F0 generation as 90% *pipiens* and 10% hybrid, and 98% *pipiens* and 2% hybrid, respectively), were unable to lay autogenous eggs and the colonies survived for only 2–4 generations. In contrast, all the other *Cx*. *pipiens* populations (ID 30, 31, 38, 41, 42, 43 and 45) laid eggs either without or after a bloodmeal supply for many generations, giving rise to well-established mosquito colonies in insectary conditions. With the exception of ID 43, which was genotyped for CQ11 as 30% hybrid and 70% *pipiens* genotypes, the other autogenous wild populations showed the CQ11 *molestus* frequency, ranging from 20% (ID 30) to 100% (ID 31, 38 and 41) and the concurrent CQ11 hybrid frequency ranging from 10% (ID 42) to 25% (ID 30).

Furthermore, to assess the genotype frequency over time, mosquito samples from a *Cx*. *pipiens* colony originating from ID 45 were analysed by CQ11 in different filial generations (Table 4).

**Table 4:** Changes in the genotype frequencies of a *Culex pipiens* colony in laboratory conditions. One hundred and fifty specimens from a laboratory-established colony (collected in Frascati, ID 45) were assayed for CQ11 after field collection, and after 7, 10 and 12 rearing generations in laboratory conditions. The percentages of the three genotypes at each generation are shown in brackets. P = *pipiens*, M = *molestus* and M/P = CQ11 hybrid.

	P	M/P	M
**F0**	8 (44.4%)	5 (27.8%)	5 (27.8%)
**F7**	4 (11.4%)	6 (17.1%)	25 (71.4%)
**F10**	11 (14.3%)	18 (23.4%)	48 (62.3%)
**F12**	1 (5%)	5 (25%)	14 (70%)

Whereas wild mosquitoes (F0) showed genotype frequencies with no statistically significant differences (Pearson *χ*^*2*^ = 1.5000; *p* = 0.472), starting from the seventh filial generation, these frequencies changed and a marked increase of the *molestus* genotype with respect to hybrid and *pipiens* genotypes was observed (Pearson *χ*^*2*^ = 36.2290; *p* ≤ 0.001). Nevertheless, no significant positive trend was found in *molestus* genotype over time (*p* = 0.488).

## Discussion

Despite the known limitations connected with the use of only one genetic locus, the CQ11 microsatellite was used for genotyping 55 Italian *Cx*. *pipiens* populations in this study. Confirming the results obtained in other similar studies [22–23,25,28–29], the CQ11 molecular assay was a valuable tool for characterising this species in the country. As the CQ11 genotyping of both wild and laboratory *Cx*. *pipiens* populations fitted with the ecological and physiological traits (commonly used to recognise the forms), there was an evidence of a genetic basis for such traits, corroborating the effectiveness of this molecular approach.

In addition, the CQ11 assay was compared with the COI assay, which has already been used to discriminate *Cx*. *pipiens* forms in the US, Russia, UK and Italy [20,26,42,51]. Although the lack of diagnostic sequence differences in the target COI region did not allow the two forms in the US *Cx*. *pipiens* populations to be recognised [42], the use of the COI assay appeared to clearly separate *molestus* and *pipiens* forms in Old World populations. In a previous entomological survey carried out in a northwestern province of Italy, the COI assay characterised all eleven populations collected in aboveground environments as *molestus*, leading to the conclusion that only this form was present in the area [51]. In the present study, this approach for Italian *Cx*. *pipiens* populations recognised both forms. Nevertheless, the molecular identification by RFLP of COI and the further sequencing did not agree with the ecological features of the populations tested, as shown by CQ11. These findings displayed an evident incongruence between CQ11 and COI assays, as was already observed by Danabalan et al. in the UK [26]. In contrast, these authors reached opposite conclusions concerning the reliability of CQ11 assay for distinguishing *Cx*. *pipiens* forms, because of the misleading presence of *Cx*. *torrentium* in their samples [26].

In this study, *Cx*. *torrentium* was not detected molecularly, but its absence is not surprising, since this species was also not found in similar surveys carried out in other Southern European countries (Southeastern France, Serbia, Greece, Turkey and Cyprus) [30], and was more frequent in Central and Northern Europe [24,27,30–32,52–53]. Nevertheless the presence of *Cx*. *torrentium* cannot be excluded in Italy, because the breeding sites of the species might occupy colder habitats at higher altitudes [17,33–34].

Only within the last few years have the bionomic and molecular data acquired concerning the distribution and composition of *Cx*. *pipiens* biotypes provided a clearer outline of the situation in Europe. As also described for other Southern European countries and North Africa [22,25,28–29,35–36], *pipiens* and *molestus* biotypes co-occur in urban, suburban, and rural habitats in Italy. Furthermore, in the majority of aboveground populations, crossbreeding of the two parental forms is a frequent event, as shown by our CQ11 genotyping results.

The reduction in heterozygosity observed in 19 *Cx*. *pipiens* populations (sample size > 18) might be due to the Wahlund effect, observed when individuals are analysed as a single mating unit but instead, belong to discrete subpopulations that do not interbreed as a whole mating unit. It can be assumed that the two forms in such localities share the same “flight habitat”, but instead of mating, prefer separate biotopes, creating substantially separate gene pools. The presence of localities which did not contain CQ11 heterozygotes (ID 13, 18 and 35) appears to confirm this supposition.

The *Cx*. *pipiens* populations that were detected exclusively in urban and underground habitats (sub-cluster D) were molecularly characterised as pure or prevalent *molestus* form populations, suggesting a marked constraint between such environments and the prevailing genotype. Previous observations have always noted that a restricted egress from hypogean breeding sites selectively favours the growth of autogenous populations, whereas underground breeding sites that readily communicate with the surrounding environment also allow the colonisation of the *pipiens* form [29,54]. These findings support our studies on the rapid adaptation of wild *Cx*. *pipiens* populations to insectary conditions, which appear to mimic a subterranean milieu.

In other Mediterranean areas, hybrids were identified in Morocco using the CQ11 assay, and the pure biotypes co-occurred in all aboveground and underground breeding sites sampled, as well as crossbreding [25]. The CQ11 locus identified both *pipiens* and *molestus* forms, and their hybrids also in Tunisia, which occurred sympatrically in different aboveground collection sites, whereas the *pipiens* biotype was not found in underground contexts [29]. In Portugal, both the CQ11 assay and microsatellite studies performed in aboveground habitats [22,28], showed a sympatric distribution of *molestus* and *pipiens* biotypes and an evident hybridisation between them. An asymmetric introgression in favour of *molestus* genes was presumed to have occurred [22]. In the North of Greece, a microsatellite approach revealed the sympatric presence of all three biotypes, with a predominance of the *pipiens* form, whereas a more genetically homogenous *molestus* biotype population was characterised in the Southern region of the country [36].

Hybridisation between the two *Cx*. *pipiens* biotypes was also sporadically observed in northern and central European countries. In Amsterdam, Reusken *et al*. [23] characterised the *Cx*. *pipiens* population in three breeding sites of underground metro stations as *molestus* (62%), *pipiens* (6.9%) and hybrid (32%) genotypes, using the CQ11 marker. A multiplex real-time PCR developed to differentiate the *Cx*. *pipiens* complex in Germany, found the *pipiens* biotype to be ubiquitous and the *molestus* biotype to widely occur in Southern regions, as well as in the Hamburg metropolitan area [27]. The analysis carried out on individual mosquito specimens from the few areas where the two forms were detected together, showed hybrids at two sites of the Rhine-Main metropolitan areas and at one site in the Hamburg metropolitan area [27]. Although a previous study carried out on the London Underground railway system using allozymes, reported that subterranean populations were genetically distinct from surface ones, with no evidence of gene flow [41], the CQ11 assay recently showed the sympatric presence of both biotypes in several aboveground breeding sites of Wales and England, which were often found together with their hybrids [26]. However, these results were not considered to be reliable by the authors, who favoured COI barcoding, which confirmed the occurrence only of the *pipiens* form in the UK [26].

These recent findings displaying the presence of hybrids in North and Central Europe suggest that the two biotypes can also interbreed at high latitudes, enabling gene flow between above- and underground populations, when the environmental conditions are suitable [9, 27, 30, 40–41, 53].

Regarding the relationship between CQ11 genotyping and phenotypic features, our analysis showed that the genetic cluster assignments were consistent with the mating and autogenic behaviour of Italian *Cx*. *pipiens* populations. Although the possibility of mating in narrow space (stenogamy) was not an exclusive prerogative of a single biotype, in every *Cx*. *pipiens* population tested, the *molestus* component, if present, became predominant in few generations, due to the ability of *molestus* males to inseminate without the need to swarm [54].

Autogeny appears to be the physiological trait that is strongly related with the CQ11^260/260^ and CQ11^260/190^ frequencies. In laboratory conditions, autogeny was established, already from the first generation, in those populations that included only CQ11 *molestus* specimens, or those together with CQ11 hybrids. In the absence of the CQ11 *molestus* fraction, autogenous ovipositions were also observed in mosquito populations that exhibited a high frequency of CQ11 hybrid genotype, as was observed for ID 43 (30% hybrid and 70% *pipiens* genotypes). On the contrary, in ID 9 and 54, which were genotyped by CQ11 as *pipiens* (90% and 98%, respectively) and showed low hybrid frequencies (10% and 2%, respectively), autogeny was totally absent and the colonies quickly declined and disappeared within a few generations. Given that autogeny is a semi-dominant character and that only a fraction of hybrids can lay eggs without a bloodmeal [55], the absence of a *molestus* fraction and/or the occurrence of very low hybrid frequencies, appear to not support an autogenous mosquito population.

## Conclusion

This study represents the first extensive molecular screening of *Cx*. *pipiens* complex in Italy. Our results show: i) the absence of *Cx*. *torrentium* at least in most of the Italian territory; ii) the ubiquitous distribution of *Cx*. *pipiens* throughout the country; iii) the simultaneous occurrence of *pipiens* and *molestus* biotypes, often in sympatry and with hybrids, both in above- and underground environments, and iv) the exclusive presence of pure *molestus* populations in hypogean environments, where the physical characteristics of the habitat hinder and completely preclude any external gene flow. These results corroborate that the CQ11 assay is a promising and robust diagnostic method for the identification of *Cx*. *pipiens* biotypes at the population level in the Palearctic Region, consistent with the ecological and physiological aspects of the populations analysed. However, taking into account the limitations connected with the use of only one molecular marker to reliably distinguish *molestus*, *pipiens* and hybrids at the individual level, a panel of microsatellite markers might be useful in the future for this purpose.

Finally, the assessment of the actual role of the three biotypes in the WNV circulation remains a crucial point to be elucidated, not only for ecological and epidemiological studies, but also for risk assessment and public health strategies. Consequently, in the light of repeated outbreaks of WND in Italy, further spatial and temporal genotyping of wild *Cx*. *pipiens* populations, together with the studies on the feeding preference and vector competence should be implemented.

## Supporting Information

S1 Table Matrix of Nei’s standard genetic distance. Pairwise values were computed for Italian *Culex pipiens* localities by Populations ver.1.2.32 software [49].
(PDF)
